# Healthy worker survivor analysis in an occupational cohort study of Dutch agricultural workers

**DOI:** 10.1007/s00420-015-1047-9

**Published:** 2015-03-21

**Authors:** E. A. J. Spierenburg, L. A. M. Smit, D. Heederik, P. Robbe, M. N. Hylkema, I. M. Wouters

**Affiliations:** 1 Division of Environmental Epidemiology, Institute for Risk Assessment Sciences, Utrecht University, Utrecht, The Netherlands; 2 Department of Pathology, University Medical Center Groningen, University of Groningen, Groningen, The Netherlands; 3 Groningen Research Institute for Asthma and COPD (GRIAC), University Medical Center Groningen, University of Groningen, Groningen, The Netherlands

**Keywords:** Healthy worker effect, Occupational exposure, Farmers, Agricultural workers, Endotoxin

## Abstract

**Objectives:**

High microbial exposures in farmers and agricultural workers are associated with less atopy. Although it has been speculated that healthy worker survival could be an explanation, this has not been studied so far. Therefore, we investigated the presence of healthy worker survival in a five-year follow-up study of an occupational cohort of Dutch farmers and agricultural industry (company) workers.

**Methods:**

We compared baseline demographic characteristics, respiratory health, atopy and endotoxin exposure of 259 workers followed up with 124 workers lost to follow-up. Additionally, baseline health status of 31 participants who had changed to lower exposure jobs at follow-up was compared to those with similar or higher exposure jobs at follow-up.

**Results:**

In general, no major healthy worker survival effect was found. Nonetheless, small differences were observed between subjects included in follow-up and those lost to follow-up. Those lost to follow-up were older, had a lower peak expiratory flow, and were less often raised on a farm. Company workers lost to follow-up with a farm childhood had more often self-reported allergy, but this was not observed for subjects with atopic sensitization or other respiratory symptoms. No differences were found for any of the studied characteristics in participants with lower exposure at follow-up compared to participants with similar or higher exposure at follow-up.

**Conclusions:**

No major healthy worker survival is present in this organic dust exposed cohort. Differences between participants lost to follow-up and participants included in follow-up with regard to health characteristics are small and unlikely to explain the previously reported inverse associations between endotoxin exposure and atopy.

**Electronic supplementary material:**

The online version of this article (doi:10.1007/s00420-015-1047-9) contains supplementary material, which is available to authorized users.

## Introduction

Healthy worker survival, a phenomenon in which workers with health problems leave the workplace, is a potential source of bias in occupational epidemiological studies. As a result of healthy worker survival, people who are sensitive to an exposure will, if possible, change their job to work either in lower exposed or unexposed jobs, while the people who are relatively less affected by the exposure would not feel the need for this change. This may lead to an underestimation of the true dose–response relationship (Stayner et al. 2003).

A decreased sensitization risk and a reduced risk for development of allergies and allergic airway problems have been linked to farm living and organic dust exposure during childhood and adulthood, while these exposures have also been shown to promote the development of non-allergic airway disease (Portengen et al. 2002; Douwes et al. 2007; Gainet et al. 2007; Thaon et al. 2011). Indeed, a protective effect of adult/occupational endotoxin exposure on hay fever and atopic sensitization and a positive association with wheeze is evident in the baseline cross-sectional data of this study cohort (Smit et al. 2008, 2010). One of the possible explanations of the inverse association of endotoxin exposure with hay fever and atopy is healthy worker survival. According to this scenario, workers exposed to high levels of endotoxin would be more likely to leave their job or move to lower exposed jobs due to preexisting allergic symptoms. Indeed, asthma has been shown to be a predictive factor in women leaving work in dairy farming, (Mounchetrou et al. 2012) and health-based selection in farmers has been proposed as an explanation for the hygiene hypothesis (van Schayck and Knottnerus 2004). However, healthy worker survival has not consistently been shown to be present in the agricultural sector (Vogelzang et al. 1999; Dosman et al. 2004; Chénard et al. 2007). A Swedish study showed that ill health was a reason for leaving work in only 1 % of famers, and farmers were shown to be healthier than the general population (Thelin and Hoglund 1994).

In industrial settings, healthy worker survival in relation to organic dust exposure has been suggested as a (partial) explanation of study results. For example, in the cotton processing industry and agricultural seed industry, exposure to organic dust has been decisively linked to (severe) inflammatory and respiratory responses (Smit et al. 2006; Shi et al. 2010) and thus a reason for possible healthy worker survival. While milder health effects have been observed in workers in the animal feed industry (Post et al. 1994), healthy worker survival based on lower lung function has also been shown to be present in the grain processing and animal feed industry (Post et al. 1998).

In this paper, we investigated the presence of healthy worker survival in our five-year follow-up study in agricultural workers. Although we do not have health and exposure information at both time points for the participants that are lost to follow-up, we do have baseline information for all participants. To investigate whether healthy worker survival occurred in our population over the time of our study, we determined whether there was a difference in baseline health and exposure status between participants included in follow-up and those lost to follow-up. This was studied both within the total cohort and as a sensitivity analysis within the subpart of the cohort that had recently started work at baseline. Additionally, in the subgroup included in follow-up, participants with lowered exposure over time were compared to those with no change or higher exposure over time.

## Methods

### Study design and population

Details of the study have been described previously (Smit et al. 2008). Briefly, the study population consisted of an occupational cohort of 901 Dutch agricultural workers recruited in 2005–2006 (baseline), of whom 525 were animal and crop farmers and 376 were agricultural industry workers (company workers). Farmers were engaged in raising cattle, pigs, chickens or goats, and/or growing fruit, vegetables or grain. The companies included flower bulb, onion, animal feed and seed processing industries (Table 1). Baseline health data were collected using a health questionnaire for all participants and a detailed health examination (details of both see below) in a subpart of the study (*n* = 453 subjects; 95 farmers and 358 company workers) (Smit et al. 2010). For logistical reasons, not all farmers were invited to participate in the health examination. Approximately five years later, the study population was approached for follow-up.

**Table 1:** Overview of the number of companies participating at baseline and those lost to follow-up per sector

Sector	All companies at baseline		Lost to follow-up		
	*n* Companies	*n* Participants (%)^a^	*n* Companies	*n* Participants (%)	Reason for not participating in follow-up
Bulbs	8	124 (100)	2	37 (29.8)	Employees not interested because of upcoming company health exam/contact person not interested
Onions	5	86 (100)	1	17 (19.8)	Big renovation going on
Animal feed	7	112 (100)	1	16 (14.3)	Company changed owner and new contact person was not interested
Seeds	3	36 (100)	0	−(0)	
Overall	23	358 (100)	4	70 (19.6)	

^a^Workers who participated in the questionnaire and health examination

Follow-up investigation consisted of the same questionnaire, lung function measurement and serum IgE analysis as baseline. To obtain this longitudinal data, farmers who previously participated in the health examination were approached by telephone. Farmers who agreed to follow-up were visited between November 2010 and May 2011. Farmers who had only filled in a questionnaire were sent a follow-up questionnaire and, if applicable, a reminder letter four months later. Companies were approached by mail, followed by a telephone call. They were told the nature of the follow-up and those who agreed to participate were visited between January and July 2011. All workers who had participated in the earlier study and had since left the company were considered lost to follow-up and were contacted by mail at their last known address to fill out a questionnaire.

In the current analysis, healthy worker survival is investigated by comparing baseline health and exposure data for those lost to follow-up and those included in follow-up. The study protocol was approved by the institutional ethics committee, and all participants gave their written informed consent.

### Questionnaire

As described previously (Smit et al. 2008), the questionnaire included items on general characteristics, farm childhood, respiratory symptoms and smoking habits. In addition, questions were included on job characteristics for company workers and job and farm characteristics for farmers. Questions on respiratory symptoms were adopted from the Dutch version of the ECRHS questionnaire (Burney et al. 1994). Asthma was defined according to the ECRHS definition as a positive response to any of the following questions: “Have you had an attack of asthma in the last 12 months?”, “Have you been woken by an attack of shortness of breath in the last twelve months?”, and “Are you currently taking any asthma medication?” Hay fever was defined as self-reported pollen allergy accompanied by itchy or watery eyes or sneezing. Wheeze was established by a positive answer to the question “Did you experience wheeze in the past twelve months?” Allergy was determined by a positive answer to the question “Have you ever had any allergies?”

### Lung function, fractional FE_NO_ and serum IgE

At baseline, a medical examination was performed consisting of a forced spirometry lung function test, blood collection and fractional exhaled nitric oxide (FE_NO_) measurements. The lung function test used a pneumotachograph and associated software (Jaeger, Würzburg, Germany) and was performed and evaluated according to European Respiratory Society standards (Quanjer et al. 1993). Age and standing height-adjusted spirometric reference values of the European Community for Steel and Coal were used (Quanjer et al. 1993). Of the lung function parameters, FEV1, FVC and PEF were used in the statistical analyses.

FE_NO_ measurements took place as previously described, on site with a Niox Mino (Aerocrine AB, Solna, Sweden) at the beginning of the workday (Smit et al. 2009).

Total and specific IgEs were measured in serum with ELISAs. Specific IgE included house dust mite, grass pollen, cat and dog allergens as previously described (Doekes 1996). Atopy was defined as positive serum IgE to one or more measured allergens. Prevalence of sensitization against cat and dog allergens was low (1.6 % and 0.7 %, respectively) and therefore not used in the analysis other than to determine atopy.

### Endotoxin exposure assessment

As described previously (Smit et al. 2008), full-shift inhalable dust samples were collected in a subset of participants and analyzed for endotoxin levels by LAL assay. Exposure was then modeled for each participant based on job description.

### Data analysis

Healthy worker survival was investigated by comparing baseline general characteristics, respiratory and allergic outcomes and endotoxin exposure levels between subjects lost to follow-up and subjects included in follow-up. FE_NO_, endotoxin exposure and total IgE were log-normally distributed and therefore log-transformed before analysis. Descriptive values given for these variables are geometric mean and geometric standard deviation.

Data were analyzed with SAS 9.2 software. Dichotomous outcomes were compared by logistic regression analysis, and continuous outcomes were analyzed by linear regression analysis. Differences in (geometric) mean levels or the prevalence between groups were considered statistically significant at *α* = 0.05. *P* values were calculated unadjusted. Adjustment for age, gender, smoking and farming childhood did not change the results (data not shown).

The main analyses were based on the subpopulation who participated both in the questionnaire and the health examination at baseline. Sensitivity analyses were performed by including subjects who filled in a questionnaire but did not participate in health examination. Additionally, an analysis stratified on farm childhood was performed as there was a significant difference in the frequency of farm childhood between those lost to follow-up and those included in follow-up, and farm childhood has previously been shown to influence endotoxin-related health effects (Smit et al. 2008).

It was assumed that in an industry with adverse respiratory or allergic health effects, healthy worker survival mostly takes place in the first years after hire (Zock et al. 1998). Therefore, an additional analysis was performed on the cohort restricted to participants who started work in the agricultural industry ≤2 and ≤5 years before the start of the baseline study. Questionnaire participants, who did not participate in the health examination, were also included in this analysis.

To investigate a possible health-motivated change in exposure level over time, a subcohort of participants included in follow-up was studied. To investigate whether healthy worker survival takes place among those remaining in the agricultural sector, participants who had changed to a job with a lower exposure at follow-up were compared to participants with similar or higher exposure at follow-up.

## Results

### Follow-up rate

**Fig. 1:**
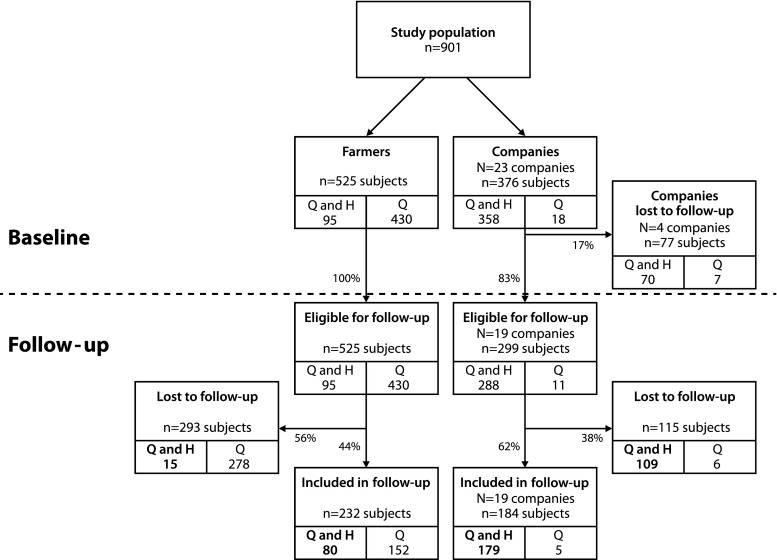
Flow chart of study population. Flow chart of study population from recruitment at baseline and eligible for follow-up till follow-up or loss to follow-up. Participants are categorized based on the information provided at baseline: questionnaire data (Q) and health exam (H). Several companies did not participate in follow-up and therefore subjects from those companies are not eligible for follow-up. For the present analysis, the main study population consists of participants who provided questionnaire and health exam information (‘Q and H’, *bold*)

A flow-scheme of the study population from baseline till follow-up is presented in Fig. 1. Nineteen out of 23 (83 %) agricultural companies participated in follow-up. The four non-participating companies had several reasons for not participating in follow-up (Table 1). The companies that were lost were divided over different sectors and attributed 70 workers (15.5 % of the initial study population); those workers were not considered to be eligible for follow-up. Of the participants eligible for follow-up and participating in the health examination at baseline, the follow-up rate was 84 % for farmers and 62 % for company workers that participated at follow-up. The follow-up rate for subjects who had only completed the questionnaire was much lower: 35 % for farmers and 45 % for company workers (Fig. 1).

For farmers who had agreed to a health examination at baseline, the main reason given for not participating in the follow-up was a lack of time or interest. No information is available on reasons for not participating in the follow-up questionnaire for farmers who had only filled in the questionnaire at baseline. Company workers were mainly lost to follow-up because they had left their original employer. The response rate to the questionnaire for those who left their job was low (*n* = 15, 7.8 %). Most of those who responded had remained in the agricultural sector and reported a change in workplace unrelated to health problems, such as termination of contract.

### Characteristics of those lost to follow-up

Participants included in follow-up resembled those lost to follow-up in the baseline general characteristics age, gender, BMI and smoking habits (Table 2). Workers included in follow-up were more likely to have grown up on a farm than were the group lost to follow-up, both in the combined analysis and in the separate company workers analysis (both *p* < 0.01). Subjects included in follow-up did not differ in baseline endotoxin exposure from those lost to follow-up in companies, farmers or the total cohort.

**Table 2:** Health and population characteristics for farmers and company workers included in follow-up (FU) or lost to follow-up (LTF) who participated in health examination at baseline

Variable	Class	Total population			Company workers			Farmers		
		*N*	Mean (SD) or *n* (%)^a^	*p*	*N*	Mean (SD) or *n* (%)^a^	*p*	*N*	Mean (SD) or *n* (%)^a^	*p*
Age (years)	LTF	124	41.85 (12.5)	0.88	109	41.55 (12.4)	0.24	15	44.03 (13.5)	0.63
	FU	259	41.68 (9.8)		179	40.02 (9.6)		80	45.39 (9.2)	
Female^b^	LTF	124	17 (13.7)	0.55	109	13 (11.9)	0.41	15	4 (26.7)	0.41
	FU	259	30 (11.6)		179	16 (8.9)		80	14 (17.5)	
BMI (kg m^−2^)	LTF	124	26.13 (4.3)	0.90	109	26.14 (4.2)	0.42	15	26.05 (4.9)	0.37
	FU	259	26.08 (3.6)		179	26.52 (3.6)		80	25.10 (3.5)	
Current smoker^b^	LTF	124	41 (33.1)	0.10	109	40 (36.7)	0.15	15	1 (6.7)	0.31
	FU	259	65 (25.1)		179	51 (28.5)		80	14 (17.5)	
Farm childhood^b^	LTF	124	36 (29.0)	<0.01	109	25 (22.9)	<0.01	15	11 (73.3)	0.28
	FU	259	148 (57.1)		179	80 (44.7)		80	68 (85.0)	
Endotoxin exposure^c^ (EU m^−2^)	LTF	124	339.00 (6.4)	0.79	109	376.77 (7.0)	0.96	15	157.34 (1.8)	0.22
	FU	259	322.36 (5.0)		179	381.34 (5.9)		80	221.34 (2.8)	
Asthma^b^	LTF	124	10 (8.1)	0.89	109	7 (6.4)	0.54	15	3 (20.0)	0.21
	FU	259	22 (8.5)		179	15 (8.4)		80	7 (8.8)	
Wheeze^b^	LTF	124	18 (14.5)	0.42	109	14 (12.8)	0.67	15	4 (26.7)	0.17
	FU	259	30 (11.6)		179	20 (11.2)		80	10 (12.5)	
Lung function (% pred.)										
FEV1	LTF	124	106.05 (14.1)	0.90	109	105.30 (14.0)	0.46	15	111.49 (14.2)	0.15
	FU	259	106.24 (15.0)		179	106.61 (15.1)		80	105.41 (15.0)	
PEF	LTF	124	113.60 (22.1)	0.03	109	114.10 (22.7)	0.04	15	109.96 (18.1)	0.21
	FU	259	118.38 (19.3)		179	119.31 (20.0)		80	116.29 (17.6)	
FVC	LTF	124	112.22 (13.4)	0.58	109	111.19 (12.9)	0.89	15	119.72 (15.3)	0.07
	FU	259	111.39 (13.9)		179	110.96 (14.0)		80	112.36 (13.9)	
FE_NO_^c^ (ppb)	LTF	123	16.20 (1.9)	0.13	108	15.71 (1.9)	0.06	15	20.25 (1.9)	0.44
	FU	258	18.07 (1.9)		178	18.29 (1.9)		80	17.58 (1.9)	
Allergy^b^	LTF	124	33 (26.6)	0.81	109	27 (24.8)	0.72	15	6 (40.0)	0.51
	FU	259	66 (25.5)		179	41 (22.9)		80	25 (31.3)	
Hay fever^b^	LTF	124	20 (16.1)	0.22	109	15 (13.8)	0.34	15	5 (33.3)	0.10
	FU	259	30 (11.6)		179	18 (10.1)		80	12 (15.0)	
TotIgE^c^ (IU/ml)	LTF	117	24.35 (9.2)	0.72	102	23.06 (9.4)	0.61	15	35.31 (7.7)	0.55
	FU	251	26.43 (7.3)		175	26.45 (8.3)		76	26.38 (5.4)	
Atopy^b^	LTF	117	22 (18.8)	0.50	102	19 (18.6)	0.35	15	3 (20.0)	0.89
	FU	251	55 (21.9)		175	41 (23.4)		76	14 (18.4)	
HDM spec. IgE^b^	LTF	117	11 (9.4)	0.30	102	8 (7.8)	0.18	15	3 (20.0)	0.49
	FU	251	33 (13.1)		175	23 (13.1)		76	10 (13.2)	
Grass pollen spec. IgE^b^	LTF	117	17 (14.5)	0.49	102	15 (14.7)	0.92	15	2 (13.3)	0.38
	FU	251	30 (12.0)		175	25 (14.3)		76	5 (6.6)	

*LTF* lost to follow-up, *FU* included in follow-up

^a^Mean (SD) for continuous variables and *n* (%) for categorical variables

^b^Categorical variable

^c^Geometric mean and SD

While subjects included in follow-up did not differ significantly from those lost to follow-up with respect to baseline self-reported asthma or wheeze, PEF was slightly lower in subjects lost to follow-up (113.6 % predicted) than in those included in follow-up (118.4 % predicted, *p* = 0.03). This was not observed for FEV1 or FVC.

Participants included in follow-up resembled those lost to follow-up in terms of allergy-related health outcomes, whether based on questionnaire data (allergy and hay fever) or on measurements (total IgE, atopy, HDM IgE and grass pollen IgE). These results did not change after adjustment for age, gender, smoking habits and farm childhood (data not shown).

The difference in prevalence of farm childhood between those lost to follow-up and included in follow-up warranted a stratified analysis by farm childhood. Because stratification on farm childhood leads to small sample sizes, especially in the group with farm childhood, the power of the analysis is limited. Sample sizes were 88/111 (LTF/FU) for no farm childhood and 36/148 for farm childhood in the total population, and 84/99 for no farm childhood and 25/88 for farm childhood in company workers. In participants who did not grow up on a farm, characteristics of those lost to follow-up were mostly the same as those included in follow-up but showed a lower PEF [*p* = 0.1 for both company workers and total cohort (Online supplement I)] and company workers had a lower BMI than those included in follow-up. Among company workers who grew up on a farm, those lost to follow-up were older (*p* = 0.03), had a higher BMI (*p* = 0.02), a higher prevalence of self-reported allergy (*p* = 0.01) and hay fever (*p* = 0.03) than those included in follow-up. This did not change when restricting the cohort to participants <60 years of age at baseline.

### Sensitivity analyses

#### Including additional subjects

A sensitivity analysis was performed by including subjects who participated by questionnaire only. This did not change the results (data not shown).

#### ≤2 and ≤5 years relevant work experience at baseline

To consider whether preferential selection takes place in our study cohort specifically in subjects who recently started work, the cohort supplemented with subjects who did not participate in health examination was restricted to subjects with ≤2 (*n* = 33) and with ≤5 (*n* = 88) years relevant work experience. Although numbers are low, in the ≤2 years subcohort atopy and HDM-specific IgE were less prevalent in participants lost to follow-up (atopy: lost = 21.4 %, included = 46.7 %, *p* = 0.02; HDM-specific IgE: lost = 7.1 %, included = 33.3 %, *p* = 0.04) and a trend for lower FE_NO_ was observed (lost = 11.9 ppb, included = 18.7 ppb, *p* = 0.07). *P* values result from analyses adjusted for age, gender and smoking. When FE_NO_ was additionally adjusted for atopy, the trend for lower FE_NO_ was no longer present (*p* = 0.20).

In the ≤5 years subcohort, participants lost to follow-up were less likely to have had a farm childhood (lost = 15.2 %, included = 35.7 %, *p* = 0.03) and tended to have both a lower PEF (lost = 106.6 %-predicted, included = 113.7 %-predicted, *p* = 0.06) and lower FE_NO_ (lost = 12.3 ppb, included = 15.2 ppb, *p* = 0.07). *P* values result from analyses adjusted for age, gender and smoking.

### Shift to lower exposure job at follow-up versus similar or higher exposure

Healthy worker survival could also have taken place as a result of participants changing to lower exposed jobs within a company, based on health problems. This possibility was investigated by comparing participants who changed to a lower exposure at follow-up (*n* = 31) to participants with the same exposure at baseline and follow-up (*n* = 113) and participants who changed to higher exposure at follow-up (*n* = 35). No significant differences or trends were found in baseline demographic characteristics, respiratory health or allergies between participants changing to jobs with lower exposure and those in jobs with similar or moving to higher exposure (Online Supplement II).

## Discussion

We investigated the possible presence of healthy worker survival in an occupational cohort of agricultural workers followed-up after five years. To this end, we conducted an analysis of the baseline data of our study supplemented with the information obtained at follow-up whether participants still worked at the same job five years later. Overall, we found no major health-related survival over the five-year time frame of this study. This seems to apply to both health-related selection resulting in leaving the workplace and to selection through changing to a job with lower exposure within a company.

We found that farmers who were lost to follow-up were not different at baseline than farmers included in the follow-up study. Previous studies in pig farmers have reported a healthy worker effect (Vogelzang et al. 1999; Dosman et al. 2004; Chénard et al. 2007). Whereas for crop and cattle farmers, little or no health-related job leaving was found (Thelin and Hoglund 1994; Mounchetrou et al. 2012). Pig farmers are known to have high endotoxin exposures (3400 ± 6.9 EU m^−3^) compared to cattle farmers (220 ± 4.6 EU m^−3^) and crop farmers (63 ± 2.2 EU m^−3^) (Smit et al. 2008). As a positive dose–response relationship between endotoxin and (respiratory) health effects is observed, stronger healthy worker survival at higher exposure is likely. The low proportion of pig farmers (3.2 % of farmers) might explain why in our total farming population no healthy worker survival was found.

The fact that we did not observe healthy worker survival in farmers does not mean there is no possibility for selection in famers at all. The size of our study population is too small to detect modest healthy worker survival effects, especially in subgroup analyses. It is also possible that a health-based selection took place outside the time frame of our study, within which we can only measure healthy worker survival. Such alternate health-related selection processes might occur gradually through generations (Bråbäck et al. 2006) or due to selection in early life such as protection through exposure during childhood (Fuchs et al. 2012) or selection at first hire (Dumas et al. 2011) where participants choose low exposure jobs to avoid related health problems. Given that the mean age of our cohort was 44 years at baseline, such selection processes could possibly have taken place prior to our study and may have resulted in a cohort consisting of workers less sensitive to organic dust, minimizing the impact of current exposure. Despite this, in our cross-sectional analyses, we observed lower prevalence of allergic endpoints and increased prevalence of respiratory symptoms with higher exposures (Smit et al. 2008, 2010). As major healthy worker survival is likely not present, this reinforces the results from our baseline cross-sectional study.

In the subpopulation of participants who worked in the agricultural industry, some differences between participants still in the study and those lost to follow-up were observed, but those are likely of minor importance biologically. Those lost to follow-up were slightly older, less often grew up on a farm and had a lower PEF. When the company cohort was restricted to those below retirement age, the difference in age disappeared, but the differences in farm childhood and PEF remained. Although the latter might indicate a slight presence of healthy worker survival, the mean PEF is still well above predicted values in the subjects lost to follow-up (114.1 %-predicted) and not likely a cause for changing jobs due to respiratory problems.

Previous studies have shown a protective effect of farm living on atopy (Ernst and Cormier 2000; Portengen et al. 2005; Basinas et al. 2012; Fuchs et al. 2012). In our previous study, having a farm childhood significantly decreased the likelihood of hay fever, wheeze and atopic sensitization but enhanced the likelihood of bronchial hyper responsiveness (Smit et al. 2008, 2010). In our present study, we looked into the differences between those who grew up on a farm and those who did not. In this lost to follow-up analysis, a difference in PEF %-predicted between participants lost to follow-up and those included in follow-up occurred only in the participants who did not grow up on a farm. Interestingly, company workers who grew up on farms and were included in follow-up differed from those lost to follow-up: the latter were older, had higher BMIs, and most noticeably reported more allergies and hay fever. These differences persisted when excluding participants close to retirement age at baseline. This might point to a preferential survival effect in subjects with a farm childhood based on health perception but not on objectively measured parameters such as specific IgE levels or lung function. As healthy worker survival is thought to be a process driven by (more or less) conscious decisions, health perception could be more important than objectively measurable health effects. However, it is unknown why such a selection would only or more often take place in subjects with a farm childhood. Finally, it should be noted that these findings might be due to low numbers in the lost to follow-up category, where an extra person with allergy or hay fever increases the percentage relatively quickly.

Selection mainly takes place early in a career (Le Moual et al. 2008). We investigated the possibility of selection in our cohort specifically in those who recently joined the agricultural industry. Interestingly, we observed that participants who were lost to follow-up in the ≤2 years’ work experience subcohort were healthier than those included in follow-up. When inclusion was changed to ≤5 years of relevant work experience, lower FE_NO_ values (a marker for lung inflammation) were found. These results suggest that people with better health are lost to follow-up, which is inconsistent with a healthy worker survival during early work experience. However, we should interpret these results with caution as the numbers involved in these subanalyses are small.

In this study, there was a high follow-up rate for workers in companies and farmers (both > 80 %). The follow-up rate was lower within participating companies (62 %). This relatively low follow-up rate made the analyses of this paper possible. Not all loss to follow-up relates to healthy worker survival. Loss to follow-up for health-related reasons could indicate healthy worker survival, while loss for other reasons like lack of time or interest might not. Like our study, most studies are not able to study healthy worker survival directly. Ideally, one would study changes in health of people over time at an individual basis and thus would be able to compare health of those who left the agricultural industry to those who stayed in. In our study, however, we could compare differences in demographics and health at baseline between subjects included in follow-up and those who are not. This still enabled us to get a good indication whether a major healthy worker selection is of importance, although the effect might be diluted due to the people that left the industry for other reasons. Because we did not find a substantial difference between participants lost to follow-up and participants included in follow-up, we can confidently state that in our cohort, there is no major healthy worker survival.

In conclusion, we show that there are no major differences in demographics and health between participants in the study and those lost to follow-up. Although pre-study selections like healthy worker hire selection cannot be excluded, a major healthy worker survival is unlikely to be present in our cohort during the time of our study.

## Electronic supplementary material

Below is the link to the electronic supplementary material.

Supplementary material 1 (DOCX 33 kb)
