# Supplementary material for: Healthy worker survivor analysis in an occupational cohort study of Dutch agricultural workers
Source: Int Arch Occup Environ Health. 2015 Mar 21;88(8):1165–73. doi: 10.1007/s00420-015-1047-9 (PMC4608974; doi:10.1007/s00420-015-1047-9)
Supplement: Supplementary file 1 — Supplementary material 1 (DOCX 33 kb) [file 420_2015_1047_MOESM1_ESM.docx]

**Online Supplement I:** Health and population characteristics of the population included in follow-up (FU) or lost to follow-up (LTF) stratified by farm childhood

|  |  | **Total population** | | | | | | | | | | |  | **Company workers** | | | | | | | | | | |
| --- | --- | --- | --- | --- | --- | --- | --- | --- | --- | --- | --- | --- | --- | --- | --- | --- | --- | --- | --- | --- | --- | --- | --- | --- |
|  |  | **No farm childhood** | | | | |  | **Farm childhood** | | | | |  | **No farm childhood** | | | | |  | **Farm childhood** | | | | |
| **Variable** | **Class** | **N** | **Mean (SD) or n (%)^1^** | | **p** | **p^2^** |  | **N** | **Mean (SD) or n (%)^1^** | | **p** | **p^2^** |  | **N** | **Mean (SD) or n (%)^1^** | | **p** | **p^2^** |  | **N** | **Mean (SD) or n (%)^1^** | | **p** | **p^2^** |
| Age [yr] | LTF | 88 | 39.75 | (12.4) | 0.79 | 0.68 |  | 36 | 46.98 | (11.4) | 0.06 | 0.06 |  | 84 | 40.00 | (12.4) | 0.33 | 0.26 |  | 25 | 46.77 | (11.3) | 0.04* | 0.03* |
|  | FU | 111 | 39.33 | (9.4) |  |  |  | 148 | 43.43 | (9.7) |  |  |  | 99 | 38.44 | (9.4) |  |  |  | 80 | 41.97 | (9.5) |  |  |
| Female^3^ | LTF | 88 | 13 | (14.8) | 0.54 | 0.53 |  | 36 | 4 | (11.1) | 0.38 | 0.34 |  | 84 | 11 | (13.1) | 0.99 | 0.99 |  | 25 | 2 | (8.0) | 0.39 | 0.19 |
|  | FU | 111 | 20 | (18.0) |  |  |  | 148 | 10 | (6.8) |  |  |  | 99 | 13 | (13.1) |  |  |  | 80 | 3 | (3.8) |  |  |
| BMI [kg.m^-2^] | LTF | 88 | 25.77 | (4.6) | 0.09 | 0.07 |  | 36 | 27.02 | (3.2) | 0.02* | 0.05 |  | 84 | 25.77 | (4.5) | 0.05 | 0.03* |  | 25 | 27.40 | (2.8) | 0.03* | 0.02* |
|  | FU | 111 | 26.81 | (4.0) |  |  |  | 148 | 25.53 | (3.2) |  |  |  | 99 | 27.04 | (4.0) |  |  |  | 80 | 25.88 | (3.0) |  |  |
| Current smoker^3^ | LTF | 88 | 34 | (38.6) | 0.30 | 0.27 |  | 36 | 7 | (19.4) | 0.91 | 1.00 |  | 84 | 33 | (39.3) | 0.26 | 0.21 |  | 25 | 7 | (28.0) | 0.76 | 0.89 |
|  | FU | 111 | 35 | (31.5) |  |  |  | 148 | 30 | (20.3) |  |  |  | 99 | 31 | (31.3) |  |  |  | 80 | 20 | (25.0) |  |  |
| Endotoxin exposure^4^ [EU.m^-3^] | LTF | 88 | 358.26 | (7.1) | 0.53 | 0.59 |  | 36 | 296.19 | (4.8) | 0.63 | 0.54 |  | 84 | 377.74 | (7.3) | 0.67 | 0.63 |  | 25 | 373.54 | (6.1) | 0.64 | 0.53 |
|  | FU | 111 | 302.58 | (6.1) |  |  |  | 148 | 338.03 | (4.2) |  |  |  | 99 | 335.02 | (6.5) |  |  |  | 80 | 447.63 | (5.2) |  |  |
|  |  |  |  |  |  |  |  |  |  |  |  |  |  |  |  |  |  |  |  |  |  |  |  |  |
| Asthma^3^ | LTF | 88 | 7 | (8.0) | 0.50 | 0.49 |  | 36 | 3 | (8.3) | 0.74 | 0.73 |  | 84 | 5 | (6.0) | 0.23 | 0.22 |  | 25 | 2 | (8.0) | 0.58 | 0.36 |
|  | FU | 111 | 12 | (10.8) |  |  |  | 148 | 10 | (6.8) |  |  |  | 99 | 11 | (11.1) |  |  |  | 80 | 4 | (5.0) |  |  |
| Wheeze^3^ | LTF | 88 | 12 | (13.6) | 0.83 | 0.92 |  | 36 | 6 | (16.7) | 0.34 | 0.37 |  | 84 | 9 | (10.7) | 0.77 | 0.72 |  | 25 | 5 | (20.0) | 0.19 | 0.17 |
|  | FU | 111 | 14 | (12.6) |  |  |  | 148 | 16 | (10.8) |  |  |  | 99 | 12 | (12.1) |  |  |  | 80 | 8 | (10.0) |  |  |
| Lung function [% pred.] | |  |  |  |  |  |  |  |  |  |  |  |  |  |  |  |  |  |  |  |  |  |  |  |
| FEV1 | LTF | 88 | 104.80 | (13.6) | 0.10 | 0.12 |  | 36 | 109.10 | (15.0) | 0.15 | 0.20 |  | 84 | 104.68 | (13.8) | 0.08 | 0.07 |  | 25 | 107.38 | (14.7) | 0.44 | 0.29 |
|  | FU | 111 | 108.03 | (13.9) |  |  |  | 148 | 104.90 | (15.7) |  |  |  | 99 | 108.30 | (13.5) |  |  |  | 80 | 104.53 | (16.6) |  |  |
| PEF | LTF | 88 | 113.63 | (21.6) | 0.01* | 0.01* |  | 36 | 113.53 | (23.7) | 0.45 | 0.47 |  | 84 | 113.65 | (21.9) | 0.01* | 0.01* |  | 25 | 115.62 | (25.3) | 0.85 | 0.89 |
|  | FU | 111 | 121.04 | (18.6) |  |  |  | 148 | 116.37 | (19.6) |  |  |  | 99 | 121.53 | (18.8) |  |  |  | 80 | 116.56 | (21.2) |  |  |
| FVC | LTF | 88 | 111.57 | (13.2) | 0.53 | 0.62 |  | 36 | 113.82 | (14.0) | 0.19 | 0.30 |  | 84 | 111.26 | (13.0) | 0.60 | 0.55 |  | 25 | 110.95 | (12.6) | 0.60 | 0.51 |
|  | FU | 111 | 112.75 | (13.4) |  |  |  | 148 | 110.37 | (14.3) |  |  |  | 99 | 112.30 | (13.6) |  |  |  | 80 | 109.29 | (14.4) |  |  |
| FE_NO_^4^ [ppb] | LTF | 87 | 15.82 | (1.9) | 0.26 | 0.33 |  | 36 | 17.16 | (2.0) | 0.55 | 0.43 |  | 83 | 15.70 | (1.9) | 0.16 | 0.26 |  | 25 | 15.73 | (2.1) | 0.27 | 0.27 |
|  | FU | 110 | 17.57 | (1.9) |  |  |  | 148 | 18.44 | (1.9) |  |  |  | 98 | 17.92 | (1.9) |  |  |  | 80 | 18.75 | (2.0) |  |  |
| Allergy^3^ | LTF | 88 | 21 | (23.9) | 0.36 | 0.39 |  | 36 | 12 | (33.3) | 0.17 | 0.10 |  | 84 | 18 | (21.4) | 0.23 | 0.26 |  | 25 | 9 | (36.0) | 0.03* | 0.01* |
|  | FU | 111 | 33 | (29.7) |  |  |  | 148 | 33 | (22.3) |  |  |  | 99 | 29 | (29.3) |  |  |  | 80 | 12 | (15.0) |  |  |
| Hay fever^3^ | LTF | 88 | 14 | (15.9) | 0.63 | 0.54 |  | 36 | 6 | (16.7) | 0.27 | 0.16 |  | 84 | 11 | (13.1) | 0.84 | 0.93 |  | 25 | 4 | (16.0) | 0.09 | 0.03* |
|  | FU | 111 | 15 | (13.5) |  |  |  | 148 | 15 | (10.1) |  |  |  | 99 | 14 | (14.1) |  |  |  | 80 | 4 | (5.0) |  |  |
| TotIgE^4^ [IU/ml] | LTF | 82 | 27.18 | (10.9) | 0.98 | 0.94 |  | 35 | 18.82 | (5.7) | 0.39 | 0.40 |  | 78 | 23.28 | (10.4) | 0.62 | 0.52 |  | 24 | 22.34 | (6.8) | 0.80 | 0.60 |
|  | FU | 106 | 26.95 | (6.7) |  |  |  | 145 | 26.05 | (7.9) |  |  |  | 95 | 27.42 | (7.2) |  |  |  | 80 | 25.35 | (9.7) |  |  |
| Atopy^3^ | LTF | 82 | 17 | (20.7) | 0.19 | 0.21 |  | 35 | 5 | (14.3) | 0.74 | 0.86 |  | 78 | 15 | (19.2) | 0.09 | 0.14 |  | 24 | 4 | (16.7) | 0.84 | 0.76 |
|  | FU | 106 | 31 | (29.2) |  |  |  | 145 | 24 | (16.6) |  |  |  | 95 | 29 | (30.5) |  |  |  | 80 | 12 | (15.0) |  |  |
| HDM spec. IgE^3^ | LTF | 82 | 10 | (12.2) | 0.46 | 0.52 |  | 35 | 1 | (2.9) | 0.17 | 0.17 |  | 78 | 8 | (10.3) | 0.22 | 0.29 |  | 24 | 0 | (0.0) | 0.97 | 0.97 |
|  | FU | 106 | 17 | (16.0) |  |  |  | 145 | 16 | (11.0) |  |  |  | 95 | 16 | (16.8) |  |  |  | 80 | 7 | (8.8) |  |  |
| Grassmix spec.  IgE^3^ | LTF | 82 | 14 | (17.1) | 0.88 | 0.98 |  | 35 | 3 | (8.6) | 0.85 | 0.75 |  | 78 | 12 | (15.4) | 0.54 | 0.72 |  | 24 | 3 | (12.5) | 0.59 | 0.54 |
|  | FU | 106 | 19 | (17.9) |  |  |  | 145 | 11 | (7.6) |  |  |  | 95 | 18 | (18.9) |  |  |  | 80 | 7 | (8.8) |  |  |
|  | |  | | | | | | | | | | | | | | | | | | | | | |  |
| ^1^ | | mean (SD) for continuous variables and n (%) for categorical variables | | | | | | | | | | | | | | | | | | | | | |  |
| ^2^ | | adjusted for age, gender, smoking and farm childhood | | | | | | | | | | | | | | | | | | | | | |  |
| ^3^ | | categorical variable | | | | | | | | | | | | | | | | | | | | | |  |
| ^4^ | | geometric mean and standard deviation | | | | | | | | | | | | | | | | | | | | | |  |

**Online Supplement II** Analysis on company workers who shifted to a different exposure at follow‑up compared to baseline and those that did not change exposure.

|  | | | **Company workers** | | | | | | | | | | | | |  |
| --- | --- | --- | --- | --- | --- | --- | --- | --- | --- | --- | --- | --- | --- | --- | --- | --- |
|  | | | **Less exposure^1^** | | |  | **Same exposure^1^** | | | |  | **Higher exposure^1^** | | | |  |
| **Variable** | | | **N** | **Mean (SD) or n (%)^2^** | |  | **N** | **Mean (SD) or n (%)^2^** | | **p^3^** |  | **N** | **Mean (SD) or n (%)^2^** | | **p^3^** |  |
| Age [yr] | | | 31 | 39.7 | (9.3) |  | 118 | 40.0 | (9.4) | 0.90 |  | 35 | 39.4 | (11.0) | 0.87 |  |
| Female^4^ | | | 31 | 1 | (3.2) |  | 118 | 16 | (13.6) | 0.15 |  | 35 | 1 | (2.9) | 0.97 |  |
| BMI [kg.m^-2^] | | | 31 | 27.3 | (3.8) |  | 114 | 26.4 | (3.6) | 0.25 |  | 34 | 26.2 | (3.6) | 0.22 |  |
| Current smoker^4^ | | | 31 | 7 | (22.6) |  | 118 | 36 | (30.5) | 0.49 |  | 35 | 8 | (22.9) | 0.94 |  |
| Farm childhood^4^ | | | 31 | 13 | (41.9) |  | 118 | 52 | (44.1) | 0.69 |  | 35 | 17 | (48.6) | 0.57 |  |
| Endotoxin exposure^5^ [EU.m^-3^] | | | 31 | 468.1 | (4.6) |  | 118 | 353.8 | (6.5) | 0.46 |  | 35 | 425.2 | (5.5) | 0.70 |  |
|  | | |  |  |  |  |  |  |  |  |  |  |  |  |  |  |
| Asthma^4^ | | | 31 | 1 | (3.2) |  | 118 | 11 | (9.3) | 0.32 |  | 35 | 3 | (8.6) | 0.36 |  |
| Wheeze^4^ | | | 31 | 4 | (12.9) |  | 118 | 12 | (10.2) | 0.61 |  | 35 | 5 | (14.3) | 0.89 |  |
| Lung function [% pred.] | | | |  |  |  |  |  |  |  |  |  |  |  |  |  |
| FEV1 | | | 31 | 104.6 | (16.6) |  | 114 | 106.8 | (14.6) | 0.35 |  | 34 | 107.8 | (15.3) | 0.36 |  |
| PEF | | | 31 | 117.2 | (17.5) |  | 114 | 119.9 | (21.0) | 0.34 |  | 34 | 119.4 | (19.0) | 0.50 |  |
| FVC | | | 31 | 108.9 | (14.7) |  | 114 | 111.3 | (13.9) | 0.33 |  | 34 | 111.7 | (13.8) | 0.36 |  |
| FENO^5^ [ppb] | | | 31 | 20.6 | (1.9) |  | 113 | 16.6 | (1.9) | 0.25 |  | 34 | 22.5 | (1.8) | 0.54 |  |
| Allergy^4^ | | | 31 | 8 | (25.8) |  | 118 | 29 | (24.6) | 0.94 |  | 35 | 5 | (14.3) | 0.24 |  |
| Hay fever^4^ | | | 31 | 4 | (12.9) |  | 118 | 11 | (9.3) | 0.64 |  | 35 | 3 | (8.6) | 0.59 |  |
| TotIgE^5^ [IU/ml] | | | 30 | 18.4 | (11.8) |  | 112 | 25.3 | (7.9) | 0.44 |  | 33 | 42.8 | (6.8) | 0.13 |  |
| Atopy^4^ | | | 30 | 6 | (20.0) |  | 112 | 26 | (23.2) | 0.46 |  | 33 | 9 | (27.3) | 0.36 |  |
| HDM spec. IgE^4^ | | | 30 | 4 | (13.3) |  | 112 | 13 | (11.6) | 0.91 |  | 33 | 6 | (18.2) | 0.51 |  |
| Grassmix spec. IgE^4^ | | | 30 | 3 | (10.0) |  | 112 | 17 | (15.2) | 0.27 |  | 33 | 5 | (15.2) | 0.42 |  |
|  | ^1^ | exposure at follow‑up compared to baseline | | | | | | | | | | | | | | |
|  | ^2^ | mean (SD) for continuous variables and n (%) for categorical variables | | | | | | | | | | | | | | |
|  | ^3^ | compared to less exposure; adjusted for age, gender, smoking and farm childhood | | | | | | | | | | | | | | |
|  | ^4^ | categorical variable | | | | | | | | | | | | | | |
|  | ^5^ | geometric mean and standard deviation | | | | | | | | | | | | | | |
